# Value of electronic alerts for acute kidney injury in high-risk wards: a pilot randomized controlled trial

**DOI:** 10.1007/s11255-018-1836-7

**Published:** 2018-03-19

**Authors:** Yanhua Wu, Yuanhan Chen, Shaowen Li, Wei Dong, Huaban Liang, Miaoyi Deng, Yingnan Chen, Shixin Chen, Xinling Liang

**Affiliations:** 1 Division of Nephrology, Guangdong General Hospital, Guangdong Academy of Medical Sciences, 106, Zhong Shan Road 2, Guangzhou City, 510080 Guangdong Province China; 2 Division of Information Management, Guangdong General Hospital, Guangdong Academy of Medical Sciences, 106, Zhong Shan Road 2, Guangzhou City, 510080 Guangdong Province China; 3 Division of Preventive Medicine, School of Public Health, Guangzhou Medical University, 195, Dongfeng West Road, Guangzhou City, 510182 Guangdong Province China

**Keywords:** Acute kidney injury, e-alert, Information system, Surveillance, Diagnosis

## Abstract

**Purpose:**

To investigate the application value of “electronic alerts” (“e-alerts”) for acute kidney injury (AKI) among high-risk wards of hospitals.

**Methods:**

A prospective, randomized, controlled study was conducted. We developed an e-alert system for AKI and ran the system in intensive care units and divisions focusing on cardiovascular disease. The e-alert system diagnosed AKI automatically based on serum creatinine levels. Patients were assigned randomly to an e-alert group (467 patients) or non-e-alert group (408 patients). Only the e-alert group could receive pop-up messages.

**Results:**

The sensitivity, specificity, Youden Index and accuracy of the AKI e-alert system were 99.8, 97.7, 97.5 and 98.1%, respectively. The prevalence of the diagnosis for AKI and expanded-AKI (AKI or multiple-organ failure) in the e-alert group was higher than that in the non-e-alert group (AKI 7.9 and 2.7%, *P *= 0.001; expanded-AKI 16.3 and 6.1%, *P *< 0.001). The prevalence of nephrology consultation in the e-alert group was higher than that in the non-e-alert group (9.0 and 3.7%, *P *= 0.001). There was no significant difference in the prevalence dialysis, rehabilitation of renal function or death in the two groups.

**Conclusion:**

The e-alert system described here was a reliable tool to make an accurate diagnosis of AKI.

## Introduction

Approximately 21% of hospitalized patients have acute kidney injury (AKI) [1]. A multicenter epidemiologic study showed that 1.4–2.9 million hospitalized patients developed AKI in China in 2013 [2]. AKI is associated with adverse outcomes. AKI may develop into chronic kidney disease (CKD) and even end-stage renal disease. AKI is associated with mortality and increased health-care costs and is a financial burden to families and societies [3, 4]. Small changes to serum levels of creatinine (sCr) are not detected by clinicians, so early intervention is unlikely [5].

In 2015, The International Society of Nephrology (ISN) proposed the “0 by 25” initiative for AKI. “Electronic alerts” (“e-alerts”) are key interventions in patients with AKI [6]. In 2016, the Acute Dialysis Quality Initiative (ADQI) stated the importance of an early diagnosis of AKI and suggested to build e-alerts to improve the diagnosis of AKI [7]. Some studies have shown that e-alerts can affect the behavior of clinicians, reduce the use of nephrotoxic drugs and improve the prognosis of AKI [8]. Wilson et al. [9] found that e-alerts for AKI did not improve clinical outcomes among hospitalized patients. The value of e-alerts for AKI is not clear. Here, we evaluated the clinical value of e-alerts for AKI patients.

## Methods

### Study design

This was a prospective, randomized, controlled study. This study is registered with clinicaltrials.gov (NCT02793167). Ethical approval of the study protocol was obtained from Guangdong General Hospital (GDREC2016164H; Guangdong, China).

The e-alert system was based on automatic measurement of sCr levels using computer software. The e-alert was based on Kidney Disease: Improving Global Outcomes (KDIGO) criteria. Patients were divided randomly into two groups: “e-alert” and “non-e-alert.” Only the e-alert group could receive pop-up windows on dashboard of the instrument.

During the study, trained researchers prospectively collected the clinical data of all patients: diagnosis upon admission and discharge from hospital; medical history; consultation records; dialysis records; final outcome in hospital.

### Interpretation and related definitions of AKI

In the present study, three classifications of AKI were used. The first was “e-alert-confirmed,” whereby the diagnosis was based on the sCr value. The second was “researcher-confirmed AKI,” whereby the researcher confirmed AKI. This type of diagnosis was considered the “gold standard” because e-alert data and clinical data were satisfied simultaneously. The final classification type was “discharge-diagnosis AKI,” which was based on medical records, and was one of the main endpoints of our study.

In this system, each sCr value could be compared with the baseline sCr value. AKI was diagnosed on the e-alert according to 2012 KDIGO-AKI guidelines [10]. The baseline sCr value was based on three rules: (1) a sCr value had been obtained in the last 2 days, and the latest value was higher by 26.5 µmol/L than the baseline sCr value; (2) a sCr value had not been obtained in the last 2 days, but a sCr value had been obtained in the last 7 days and, using the lowest value as the baseline, the latest sCr value was 50% higher than the baseline sCr value; (3) a sCr value had not been obtained in the last 7 days, but a sCr value had been obtained in the last 30, 90 or 365 days; using the lowest sCr value in the above time range as the baseline, the latest sCr value was 50% higher than the baseline sCr value. The AKI stage was classified as 1, 2 or 3 according to the maximum value of sCr (Table 1).

**Table 1:** Staging of AKI

Stage	Maximum sCr value
1	1.5–1.9 times baseline OR ≥ 0.3 mg/dL (≥ 26.5 µmol/L) increase
2	2.0–2.9 times baseline
3	3.0 times baseline OR increase in serum creatinine to ≥ 4.0 mg/dL (≥ 353.6 µmol/L) OR initiation of renal replacement therapy

The clinical data of all cases were collected prospectively by trained researchers. Researchers confirmed AKI after ruling out the following conditions in the e-alert: (1) baseline sCr > 353.6 µmol/L; (2) a history of stage-5 chronic kidney disease (CKD) or maintenance hemodialysis; (3) kidney transplantation; (4) amputation; (5) no clinical evidence to support a diagnosis of AKI.

For non-e-alert patients, the researchers screened for AKI-related disease processes (e.g., oliguria) and confirmed them to be non-AKI. The sensitivity, specificity, Youden Index (sensitivity + specificity − 1) and accuracy of e-alerts were calculated using researcher-confirmed AKI as the gold standard.

Discharge-diagnosis AKI was judged according to the *International Classification of Disease* (tenth revision, clinical modification, ICD-10). Relevant codes were N17 and N10 x00 (acute renal tubular interstitial nephritis), N14.102 (contrast nephropathy), N18.80001 (acute exacerbation of CKD), N99.000 (kidney failure after operation) and N99.001 (kidney failure after surgery). Two ICDs for multiple-organ dysfunction syndrome (MODS) were included in discharge-diagnosis AKI: R65.101 (infectious multiple-organ dysfunction syndrome) and R65.301 (multiple-organ dysfunction syndrome). The diagnostic prevalence of AKI and expanded-AKI was obtained by calculating the ratio of discharge-diagnosis AKI and researcher-confirmed AKI.

The recovery of kidney function among discharged patients was defined as shown in Table 2.

**Table 2:** Recovery of kidney function among patients discharged from hospital

Recovery of kidney function	sCr at discharge
Total	< 1.2 times of baseline sCr
Partial	≥ 1.2 times and 1.5 times of baseline sCr
None	≥ 1.5 times of baseline sCr or dependent upon renal replacement therapy

### Development of the e-alert system for AKI

The e-alert system was developed by the divisions of Nephrology and Information Management of Guangdong General Hospital (Patent Application 201610001950.5). The e-alert system consisted of a patient filter, sCr extractor, AKI automatic interpretation, random-number generation and distribution, and pop-up window generator. The system generated the random allocation sequence according to the time of acute kidney injury.

**Fig. 1:**
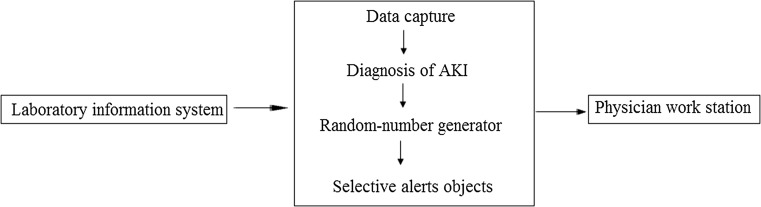
Structure of the electronic-alert system for AKI

The working principle of the e-alert system was ingenious. First, the system screened adult patients aged ≥ 18 years who could be alerted. Upon hospitalization, the system would compare the sCr values (including the results of previous hospitalizations or outpatient visits). Then, the system made a diagnosis of AKI according to KDIGO criteria. Based on the generation of random numbers, the system divided patients into an e-alert group and non-e-alert group. Only the e-alert group would receive pop-up windows (Fig. 1). The participants were blinded after assignment to interventions.

### Operation of the e-alert for AKI

The e-alert system was run in intensive care units (ICUs), cardiology divisions (eight divisions), coronary care units (CCUs) and cardiac surgery divisions (four wards) in Guangdong General Hospital from July 1 to November 31, 2016.

### Statistical analyses

Analyses were done using SPSS v20.0 (IBM, Armonk, NY, USA). Continuous data are presented as the mean (standard deviation) or median (interquartile range) as appropriate, and categorical variables as *n* (%). We compared groups using the Student’s *t* test for continuous variables and *χ*^2^ test for categorical variables. For ordered classification variables and non-normally distributed measurement data, we used the rank sum test. All tests were two-tailed, and *P *< 0.05 was considered significant.

## Results

### General information

**Fig. 2:**
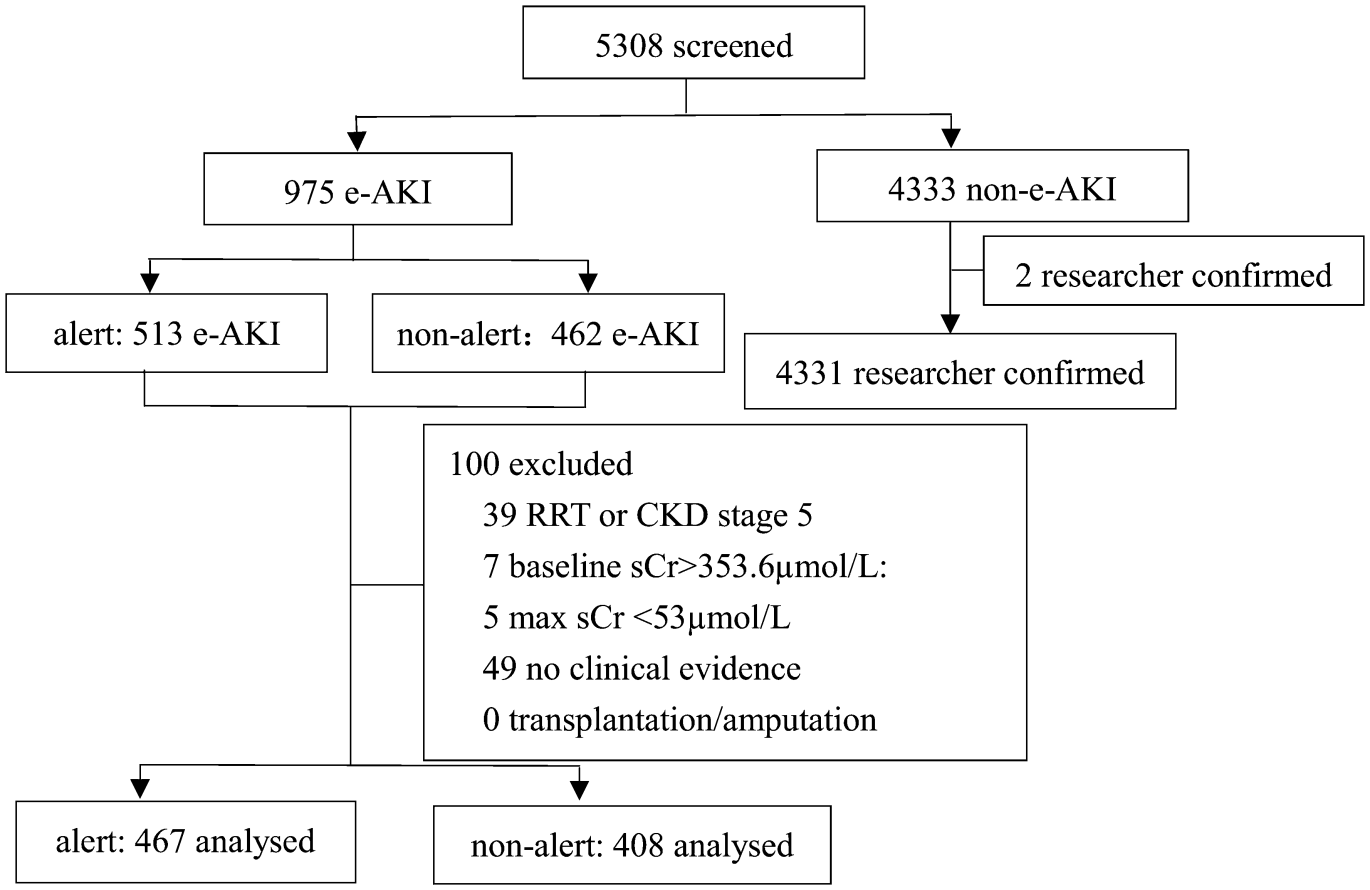
Trial profile

During the operation of the e-alert system, 5308 patients were screened in our hospital. There were 975 (18.3%) e-alerts according to 2012 KDIGO criteria. At random, 513 patients received an e-alert, and the remainder (462 patients) did not. According to the exclusion criteria, 467 patients (in the e-alert group) and 408 patients (in the non-e-alert group) were researcher-confirmed AKI. From 4333 non-e-alert patients, clinical evidence (e.g., oliguria) was used to diagnose AKI in two patients (Fig. 2).

There was no significant difference between the e-alert group and non-e-alert group with regard to sex, age, age group, baseline sCr value or Charlson score (Table 3).

**Table 3:** Baseline characteristics of study participants

	e-alert group (*n* = 467)	Non-e-alert group (*n* = 408)	*t*/*χ*^2^/*Z*	*P*
Sex			0.535	0.465
Male (%)	298 (63.8)	270 (66.2)		
Female (%)	169 (36.2)	138 (33.8)		
Age (year) [median (Q25, Q75)]	62 (53–71)	64 (54–72)	− 1.713	0.087
Age group (years)			4.982	0.173
18–39 (%)	40 (8.6)	21 (5.1)		
40–59 (%)	148 (31.7)	126 (30.9)		
60–79 (%)	229 (49.0)	221 (54.2)		
≥ 80 (%)	50 (10.7)	40 (9.8)		
Baseline sCr (µmol/L) [median (Q25, Q75)]	82.0 (57.3–111.0)	79.5 (58.0–111.0)	− 0.505	0.614
Charlson score	2 (1–3)	2 (1–3)	− 0.112	0.911

### Authenticity of e-alerts

Using researcher-confirmed AKI as the gold standard, we evaluated the authenticity of the e-alert system for AKI. The total number of e-alerts was 976, of which 875 were researcher-confirmed AKI. The total number of non-e-alerts was 4333, of which 2 were researcher-confirmed AKI. Evaluation of e-alerts revealed the sensitivity to be 99.8%, specificity to be 97.7%, Youden Index to be 97.5% and accuracy to be 98.1%.

### Value of e-alerts for improving the prevalence of AKI diagnosis

The prevalence of the diagnosis of AKI in the e-alert group was higher than that for the non-e-alert group, and the difference was significant (7.9 and 2.7%, *P* = 0.001). The prevalence of the diagnosis of expanded-AKI was also higher in the e-alert group than that in the non-e-alert group, and the difference was significant (16.3 and 6.1%, *P* < 0.001). In patients with stage-1 AKI, the prevalence of the diagnosis of AKI and expanded-AKI was higher in the e-alert group than in the non-alert group (AKI: 3.5 and 0.7%, *P *= 0.048; expanded-AKI: 7.7 and 1.8%, *P* = 0.003). However, for stage-2 and stage-3 AKI, a significant difference was not observed in the two groups (AKI: *P* = 1.000, 0.181; expanded-AKI: *P* = 0.555, 0.306). In a ward-stratified study, e-alerts improved the prevalence of the diagnosis of AKI and expanded-AKI in cardiology divisions (AKI: 5.0 and 0.0%, *P* = 0.011; expanded-AKI: 8.3 and 0.0%, *P* < 0.001) and cardiac surgery divisions (AKI: 9.0 and 2.7%, *P* = 0.019; expanded-AKI: 17.5 and 6.8%, *P* = 0.004). There was no significant difference in the ICU or CCU (AKI: *P* = 0.298, 0.714; expanded-AKI: *P* = 0.091, 0.349) (Table 4).

**Table 4:** Relationship between the prevalence of the diagnosis of AKI in e-alert and non-e-alert groups

	AKI		*χ* ^2^	*P*	Expanded-AKI		*χ* ^2^	*P*
	e-alert (*N* = 467)	Non-e-alert (*N* = 408)			e-alert (*N* = 467)	Non-e-alert (*N* = 408)		
Total prevalence (%)	37 (7.9)	11 (2.7)	11.474	0.001	76 (16.3)	25 (6.1)	21.957	< 0.001
*Stage*								
1 (%)	5 (3.5)	2 (0.7)	–	0.048	11 (7.7)	5 (1.8)	8.982	0.003
2 (%)	9 (4.6)	4 (5.1)	–	1.000	22 (11.3)	7 (8.9)	0.348	0.555
3 (%)	23 (17.8)	5 (9.8)	1.792	0.181	43 (33.3)	13 (25.5)	1.049	0.306
*Division*								
ICU (%)	11 (9.0)	4 (5.1)	1.085	0.298	27 (22.1)	10 (12.7)	2.865	0.091
Cardiology (%)	6 (5.0)	0 (0.0)	–	0.011	10 (8.3)	0 (0.0)	–	< 0.001
CCU (%)	4 (8.5)	3 (6.2)	–	0.714	8 (17.0)	5 (10.4)	0.877	0.349
Cardiac surgery (%)	16 (9.0)	4 (2.7)	5.535	0.019	31 (17.5)	10 (6.8)	8.336	0.004

### Relationship between e-alerts and outcome

The prevalence of nephrology consultation in the e-alert group was higher than that in the non-e-alert group (9.0 and 3.7%, *P *= 0.001). There was no significant difference in the prevalence of dialysis, rehabilitation of renal function or death in the two groups (*P* = 0.885, 0.382 and 0.160) (Table 5).

**Table 5:** Relationship between e-alerts and outcome

	Alert (*N* = 467)	Non-alert group (*N* = 408)	*χ*^2^/*Z*	*P*
Consultation (%)	42 (9.0)	15 (3.7)	10.109	0.001
Renal replacement therapy (%)	46 (9.9)	38 (9.6)	0.021	0.885
Total outcome in hospital (%)			3.061	0.382
Death (%)	42 (9.0)	33 (8.1)		
Discharge without order (%)	33 (7.1)	24 (5.9)		
Transfers with order (%)	5 (1.1)	1 (0.2)		
Discharge with order (%)	387 (82.9)	350 (85.8)		
Renal-function outcomes among patients discharged with order			3.665	0.160
Totally recovery (%)	223 (57.6)	213 (60.9)		
Partially recovery (%)	97 (25.1)	94 (26.9)		
Non recovery (%)	67 (17.3)	43 (12.3)		

## Discussion

AKI is a common and serious clinical syndrome, especially in the ICU. Due to the insufficiency of cardiac function, the use of contrast agents and extracorporeal circulation in cardiac surgery, [10] cardiology divisions become the “frontline” in the battle against AKI.

The ISN and ADQI suggested that e-alerts should be constructed to resolve the early diagnosis of AKI. However, Wilson et al. [9] found that e-alerts did not improve the clinical endpoints of patients with AKI. One of the limitations they considered was that the role of e-alerts in different wards might be different. Therefore, we applied e-alerts to the high-risk wards of AKI and evaluated its value.

An accurate and timely diagnosis is prerequisites for effective interventions. In ICUs, if AKI or other types of organ dysfunction occur, clinicians are more likely to diagnose MODS. Hence, we included MODS in the diagnosis of expanded-AKI. The prevalence of the diagnosis for AKI and expanded-AKI was higher in the e-alert group than that of the non-alert group. In 2013, AKI was documented in 1.4–2.9 million hospitalized patients [2]. The e-alert used in the present study increased the prevalence of the diagnosis of AKI from 2.7 to 7.9%. This means that it can reduce the missed diagnosis of 72,800–150,800 cases nationwide per year in China.

Research has shown that even the clinical manifestations of low-level AKI or “transient” AKI are associated with dialysis, cardiovascular disease and death [11]. In the present study, e-alerts were more effective in improving the prevalence of the diagnosis of patients with stage-1 AKI. AKI can occur in different hospital divisions. The AKI-related knowledge of physicians in different specialties is not optimal, especially in non-ICU and surgery divisions. The preliminary results of our study showed that e-alerts can improve the prevalence of the diagnosis in cardiology and cardiac surgery divisions.

In the present study, use of e-alerts reduced the prevalence of the missed diagnosis of AKI, but the overall prevalence was very low. There was no improvement in the prevalence of dialysis or renal-function recovery in survivors. There could have been three main reasons for this observation. First, the high prevalence of AKI in high-risk wards was not matched with monitoring of renal function, especially in non-ICU and surgery wards. Second, some clinicians did not understand the meaning of e-alerts. This information system is convenient for medical management, but, with increasing use of e-alerts for different diseases, “e-alert fatigue” may occur [12]. Third, nephrologists did not intervene actively. Early intervention and follow-up by nephrologists can improve the prognosis of AKI [13]. An alert system alone is not adequate to improve the effectiveness of AKI management. Therefore, it is necessary to strengthen the education of clinicians. Through meetings and lectures, we can explain the purpose, importance and methods of the present study.

There were two study limitations. First, we lacked follow-up data such as long-term rehospitalization and other adverse outcomes. Second, because preadmission and in hospital medical records are not combined in most Chinese hospitals, it is difficult to distinguish community-acquired AKI with e-alert.

The present study evaluated, in a preliminary fashion, the value of application of e-alerts in AKI high-risk wards. e-alerts reduced the prevalence of a missed diagnosis of AKI to some extent, but there was no improvement in the main endpoints. Our research team intends to strengthen the training of physicians in relevant divisions of our hospital. In the future, we will reevaluate the value of e-alerts based not only on the inhospital outcomes, but also on long-term prognosis.
